# Alu-element insertion in an *OPA1* intron sequence associated with autosomal dominant optic atrophy

**Published:** 2010-02-10

**Authors:** Gian Nicola Gallus, Elena Cardaioli, Alessandra Rufa, Paola Da Pozzo, Silvia Bianchi, Camilla D’Eramo, Michele Collura, Manuela Tumino, Lorenzo Pavone, Antonio Federico

**Affiliations:** 1 Department of Neurological, Neurosurgical and Behavioural Sciences, University of Siena, Siena, Italy; 2 Italian Union of the Blind, Siracusa, Italy; 3 Department of Pediatric, University of Catania, Catania, Italy

## Abstract

**Purpose:**

Autosomal dominant optic atrophy (ADOA) is the most common form of hereditary optic neuropathy caused by mutations in the optic atrophy 1 (*OPA1)* gene. It is characterized by insidious onset with a selective degeneration of retinal ganglion cells, variable loss of visual acuity, temporal optic nerve pallor, tritanopia, and development of central, paracentral, or cecocentral scotomas. Here we describe the clinical and molecular findings in a large Italian family with ADOA.

**Methods:**

Routine ophthalmologic examination and direct sequencing of all coding regions of the *OPA1* gene were performed. Further characterization of a new *OPA1* gene insertion was performed by reverse transcription-PCR (RT–PCR) of RNA from patients and control subjects.

**Results:**

We identified an Alu-element insertion located in intron 7 of *OPA1* causing an in-frame deletion of exon 8 in 18 family members.

**Conclusions:**

The predicted consequence of this mutation is the loss of the guanosine triphosphatase (GTPase) activity of OPA1. Alu insertions have been reported in the literature as causing human genetic disease. However, this is the first report of a pathogenic *OPA1* gene mutation resulting from an Alu insertion.

## Introduction

Autosomal dominant optic atrophy (ADOA; OMIM 165500) is the most common form of hereditary optic neuropathy, with an estimated prevalence between 1:10,000 and 1:50,000 in different populations [1]. It is characterized by insidious onset with a selective degeneration of retinal ganglion cells, variable loss of visual acuity, temporal optic nerve pallor, tritanopia, and development of central, paracentral, or cecocentral scotomas [2]. ADOA is inherited in an autosomal dominant manner with high interfamilial and intrafamilial phenotypic variability and incomplete penetrance.

Mutations in the optic atrophy 1 (*OPA1)* gene (that consists of 30 coding exons) are responsible for approximately 90% of cases. *OPA1* encodes a large guanosine triphosphatase (GTPase), implicated in the formation and maintenance of the mitochondrial network [3] and in protection against apoptosis by segregating cytochrome *c* inside the mitochondrial cristae [4]. OPA1 comprises a highly basic N-terminus, a dynamin GTPase domain, and a C-terminus. More than 200 pathogenic mutations have been reported in the literature (see the eOPA1 database). No significant correlation has been observed between degree of visual impairment and location or type of mutation [5]. Missense mutations in *OPA1*, however, leading to multiple mitochondrial DNA (mtDNA) deletions in skeletal muscle and a mosaic defect of cytochrome *c* oxidase, were recently found. In these cases the disorder presented with visual failure and optic atrophy in childhood, followed by Progressive External Ophthalmoplegia (PEO), ataxia, deafness, and sensory-motor neuropathy in adulthood. Moreover, some cases show additional neurologic symptoms, the so-called optic atrophy *plus* phenotypes [6].

## Methods

### Family study

**Figure 1:**
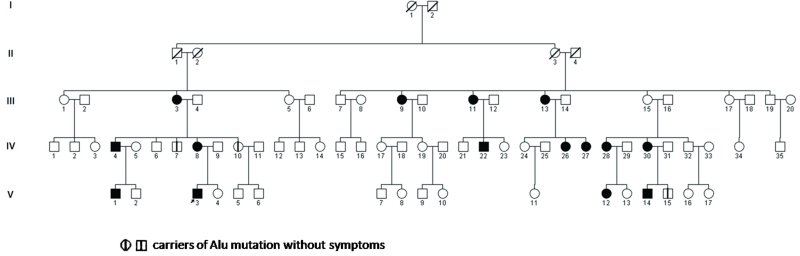
Pedigree diagram of the Autosomal Dominant Optic Atrophy (ADOA) family. The pedigree of a five-generation family shows eighteen affected members. Solid squares mean male patients, open circles mean normal females, solid circles mean female patients, open squares mean normal males, arrow means proband (V-3). Oblique lines through circles and squares represent deceased, vertical lines indicate the presence of the mutation in two unaffected family members (IV-7 and IV-10) and in another family member not available for ophthalmological examination (V-15).

Twenty-eight individuals of a single large Italian family were recruited from the Department of Neurologic, Neurosurgical and Behavioral Sciences of Siena, the Italian Union of the Blind of Siracusa and the Department of Pediatric of Catania. There were 15 females and 13 males ranging in age from 9 to 59 years. We use the term “family” to indicate descendants of a multigenerational pedigree of related individuals (Figure 1). Autosomal dominant inheritance was indicated by multiple affected individuals in each generation. All patients underwent routine ophthalmologic examination, including visual acuity, color vision, manual visual field, slit-lamp examination, intraocular pressure (IOP), and dilated fundoscopy. Clinical neurologic examination and genetic analysis was also performed in all patients.

### Mutation analysis

After informed consent was obtained, 5 ml of blood from the proband, his family members, and healthy controls, was drawn into an ethylenediamine tetraacetic acid (EDTA) sample tube and preserved (frozen). Genomic DNA was extracted from blood samples using a QIAamp DNA blood kit (Qiagen, Limburg, Netherlands). All 30 coding exons (comprising intron–exon boundaries) were amplified by PCR using primers specific for *OPA1* (Table 1). PCR reactions were performed in 25 μl reaction volumes containing 100 ng genomic DNA, 10 pmol forward and reverse primers, 100 mM dNTPs, 1U DNA polymerase (Eurotaq DNA polymerase; Euroclone, Milan, Italy), 1.5 mM MgSO_4_, 1× Reaction buffer, using a DNA thermal cycler (PTC-200; MJ Research, Waltham, MA). The PCR products of exon 8 were cloned into a PCR Cloning Vector pSC-A using the Strata Clone™ PCR Cloning Kit (Agilent Technologies, Stratagene Products Division, Santa Clara, CA). After purification of PCR products with QIAquick PCR Purification kits (Qiagen), sequencing was performed using the automated sequencer ABI 3730 (Applied Biosystems, Foster City, CA).

**Table 1:** DNA sequence of oligonucleotides used for polymerase chain reaction (PCR).

**Amplified fragment**	**Forward primer (5′-3′)**	**Reverse primer (5′-3′)**	**PCR length (bp)**	**Annealing temperature °C**
Promotor	CATGGCTAGTACGGATGTAATT	GGAGAAACTAACCACCATCA	250	55
Exon 1	TGTTTCCGTGACGGACTGAGTA	CCTGAGAGTCACCTGCACATTT	330	56
Exon 2	TCCTTTGTACTGTTACCCTCTC	CTAGTCCATGGTAGAGACACAA	530	54
Exon 3	CATTATTCTTAGTAGATTAATGTG	AAACAGTTAGTGGCAGCTGTGG	340	56
Exon 4	GTAGGGTTGTCATGAGGATTAA	CCTCCATGGTTCTTAAATTT	270	54
Exon 4b	GAAATCTGAGATCTCAGACATC	TGGTCTGCAATTTTATTTGCAAC	250	57
Exon 5	TCAGCTTAGGCTGTTGACATCAC	CCAGAACTGCCACGTAATACCT	300	62
Exon 5b	TCCCTAGCTTACATCTGTTCCT	TGGCAGAAATGGCTGATTAGTG	270	57
Exon 6	GCAACAGGGAAATGATTGCCTT	AGGTTCTATCATTACACCTTCC	410	56
Exon 7	AGGAGATATGACTTCAAGATTTTGG	CCATCCTCCAAGCACATTAGGTT	350	57
Intron 7	AGGAGATATGACTTCAAGATTTTGG	CATGTTTGTTAAGCTAGAACTG	900	56
Intron 7	CTGCAGTAAATCATGGCATGT	GAGAGAGAAAGAGAGAGGACAG	650	52
Intron 7	CAGTGGATACTTAACCACCC	GAGAACATCAAGAACTTCAG	450	54
Exon 8	CTCTTGACACATCTGTTATATT	GCCTACATCTAGTATGTATA	270	52
Exon 9	CTCAGAGCAGCATTACAAATAGG	AACACAATGAACAGGTCTCACTG	250	57
Exon 10	CAATGCAGTAGCCCTGTCTAGA	GGCTAACGGTACAGCCTTCTTT	380	62
Exon 11	GATAGTTCTCGGGAGTTTGATC	GCTCTACATATCTAGATAGCAGC	360	62
Exon 12 and 13	GTCTTATCTGAATGGATGAG	GCAAAGCTTGGATTGCTAAAGAAG	420	57
Exon 14	CTTGCTATAATGTAGACACAGGG	AAATCCCTATCACAGCTGGAGC	300	62
Exon 15 and 16	TAGGCAGCAGAGCAGGAATTCA	CAGTTCAATTTAAGCTACTCTC	400	56
Exon 17	AGCACATTCGCAGACTTGGTGGTA	GTATGGATGCCAAAGATTGCCAGC	260	68
Exon 18	CCACTTTAACCACTACATCTGG	GAGATAACTGCTCCTAGAGATG	300	62
Exon 19	CCTCCCTTTGGTTATCTCTGAA	GCCTATGAGCCAAGGCAACAATAAAT	270	54
Exon 20	CTTGACTGGTGCGATTTACAGG	GAAAGATAGAGGCTGTGATGGG	430	60
Exon 21	CTGTTTGGCTTGAGCTCGTGTT	TGAGGCTGATACCCCAGTATAC	430	62
Exon 22	GGGCCAGGAGAGAATCTCCACT	TGGATGACTCCTTCACCACTGT	350	62
Exon 23	CTGCCTTCATATTGATATAGCAC	ATTCCTGAGACTGGTCTAGAGC	300	54
Exon 24	TTTACCATATTCATAAGCCGGG	TGTCAGCAAATGTACACGTGAC	450	60
Exon 25	CTTCTCAGTGTGGTTGATCAAC	CCCCAGATGATCAAAGGACTTA	300	50
Exon 26	CTGGTTCTAGTAGTTGTATGTG	AGCTACCACACCTGGCCAGTTT	350	56
Exon 27	GCACTTCCATTAAAGTGTATAGC	ACAAATGGAAATGGGAAAGGTGG	420	62
Exon 28	AAGGTGGTATGGTGAGACTCAT	TACAGCATAAGTGACAAGCAGG	350	62

### Reverse Transcriptase–PCR analysis

Peripheral blood, about 10 ml, was collected using vacutainer EDTA tubes (Becton-Dickinson, Franklin Lakes, NJ) from the proband (V-3, Figure 1), the proband’s mother (IV-8, Figure 1) and three healthy controls. Total RNA was extracted from peripheral blood leucocytes using the LeukoLOCK™ Total RNA Isolation System following the instruction supplied by the manufacturer (Ambion, Austin, TX). The amount of total RNA extracted from each blood sample was quantified using the NanoDrop ND-1000 photometer (NanoDrop Technologies, Wilmington, DE). For each sample, 1.0 μg of total RNA was reverse transcribed with Affinity Script (Agilent Technologies) using oligo dT primers. PCR amplifications were performed with primers AF1 (5′-GAC CAA CTT CAG GAA GAA CT-3′) and AR1 (5′-TGA GGA CCT TCA CTC AGA GT-3′). After the purification of cDNA products with QIAquick PCR Purification kits (Qiagen), sequencing was performed using the automated sequencer ABI 3730.

## Results

### Clinical findings in carriers of mutations

Neurologic examination was unremarkable (Table 2). Ophthalmologic examination revealed visual acuity ranging from 20/20 to less than 20/200. Color vision was normal in two subjects, the other 16 had different degrees of color sensitivity deficiency (eight of them showed generalized color sensitivity deficit). IOP with applanation was in the normal range in 11 patients (not exceeding 19–20 mmHg); four subjects had IOP over 20 mmHg. One patient had high IOP (40 mmHg). Optic nerve appearance was normal in two subjects, whereas nine showed temporal pallor and six diffuse neurorim pallor.

**Table 2:** Patients clinical findings.

**Case**	**Age**	**Visual acuity**		**Color vision Ishihara**			**IOP applanation (mmHg)**		**Ophthalmoscopy**	
** **	** **	**RE**	**LE**	**RE**		**LE**	**RE**	**LE**	**RE**	**LE**
III-3	59	20/40	20/50	2/15			18		diffuse neurorim pallor C/D 0.3	
III-9	58	<20/200		generalized deficit			16		temporal pallor C/D 0.6–0.7	
III-11	56	<20/200		generalized deficit			20		optic atrophy C/D 0.6–0.7	
III-13	51	<20/200		generalized deficit			14		temporal pallor C/D 0.6–0.7	
IV-4	41	<20/200	20/15	0		3/15	40	12	optic atrophy C/D 0.6	subatrophy C/D 0.4
IV-7	20	20/20		15/15			21		>cupping C/D 0.5–0.6	
IV-8	38	20/20		12/15		15/15	19	22	slight temporal pallor C/D 0.5–0.6	cupping CD0.6–0.7
IV-10	36	20/20		15/15			normal		normal	
IV-22	33	20/40	20/70	generalized deficit			14		temporal pallor C/D 0.6–0.7	
IV-26	25	<20/200		generalized deficit			16		temporal pallor C/D 0.3–0.4	
IV-27	27	<20/200		generalized deficit			16		temporal pallor C/D 0.3–0.4	
IV-28	41	20/70		4/15			16	17	diffuse neurorim pallor C/D: 0.4	
IV-30	48	20/70	20/100	generalized deficit			15		normal cupping	
V-1	10	20/40		4/15			np		temporal pallor C/D 0.3	
V-3	9	20/100		generalized deficit			np		temporal pallor C/D 0.7	
V-12	12	20/20		15/15			normal		slight temporal pallor. Normal cupping	
V-14	17	20/50		12/15	10/15		18		diffuse neurorim pallor	
V-15	21	N.A.		N.A.			N.A.		N.A.	

The phenotypic features of seventeen family members with *OPA1* mutation are shown. The numbering of patients in the first column is the same as in Figure 1. The second column indicates age at diagnosis (in years). The fourth column indicates results of the Ishihara color test (a test for color blindness). The fifth column indicates intra-ocular pressure with applanation. Abbreviations: C/D represents cup to disk ratio; IOP represents intra-ocular pressure; RE represents right eye; LE represents left eye; N.P. represents not performed; N.A. represents not available.

### Molecular studies

**Figure 2:**
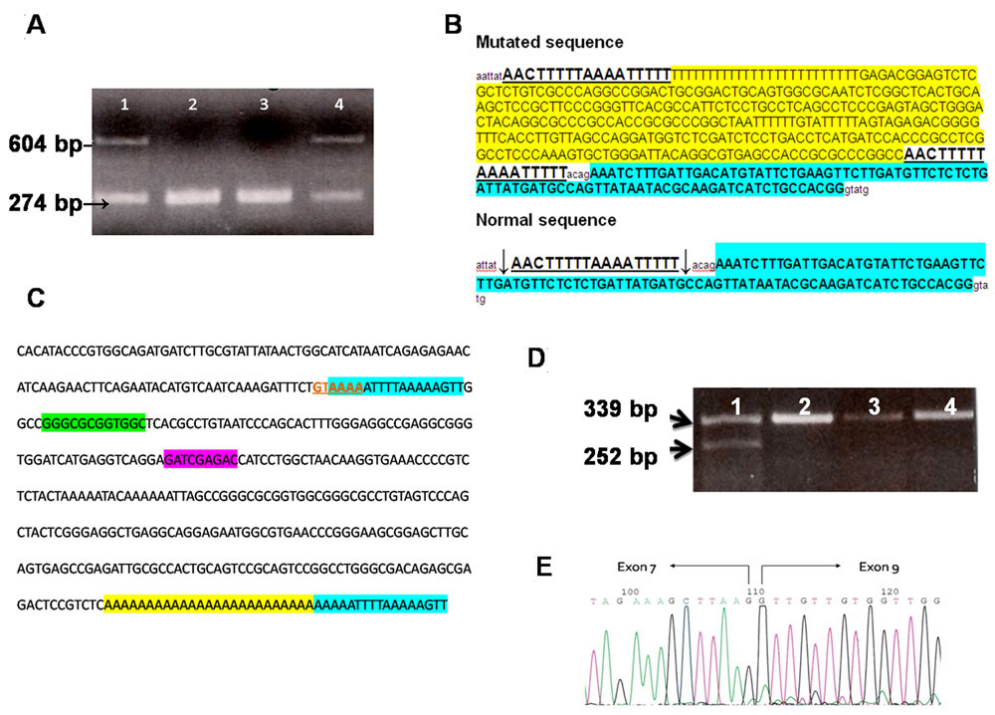
*OPA1* mutation in the Autosomal Dominant Optic Atrophy (ADOA) family. **A** shows the agarose gel of products from polymerase chain reaction (PCR) of exon 8. Lane 1 is patient sample (V-3), lanes 2 and 3 are normal controls, lane 4 is patient’s mother (IV-8). **B** shows comparison of sequences of allele with Alu insertion and normal allele. Exon sequence is shown in bold and highlighted in sky blue; direct repeats flanking the Alu insertion are shown in bold and underlined; Alu element with poly(A) tail is highlighted in yellow; arrows in the normal sequence indicate the cuts. **C** shows complementary and reverse sequence of mutated region in *OPA1* gene with sequence elements of the Alu repeat. Consensus target site is shown in red bold characters and underlined; target-site duplications of the *OPA1* gene sequence flanking the integrated DNA are highlighted in sky blue; A and B box sequences (RNA polymerase III promoter) are highlighted in green and violet, respectively; poly(A) tail is highlighted in yellow. **D** shows the agarose gel of products from reverse transcription–PCR of exons 6–10 of the *OPA1* gene. Lane 1 is patient sample (V-3), lanes 2, 3, and 4 are normal controls. **E** shows electropherogram section with the skipping of exon 8.

We sequenced the 30 coding exons of *OPA1* in the propositus. We did not find any known pathogenic mutation, but amplification of exon 8 revealed a hemizygous product of about 604 bp (Figure 2, A). Cloning and sequencing of the longer band revealed an insertion of about 325 bp. Using BLAST and Repeat Masker [7] to elucidate the nature of the inserted fragment, we identified 289 bp of the insert as belonging to AluYb8 (99.65% homology with Alu Sb2). The Alu insert was composed of a 289-bp fragment and contained a 25-bp poly(A) tail at its 3′ end with perfect direct repeats of 17 bp flanking the insert (Figure 2B,C). In Figure 2C RNA polymerase III promoter (A and B boxes) and the consensus target site sequence are also indicated. The same mutation was found in 17 family members (Figure 1); in ten subjects the mutation was absent, and the analysis was not performed in the remaining family members. The Alu insertion was located in intron 7 of *OPA1*, 21 bp upstream of exon 8, near the acceptor site (C.784–21_22). The mutation caused the skipping of exon 8 in mRNA (Figure 2D,E).

## Discussion

In the case reported here, we found an unusual molecular defect in *OPA1* causing ADOA in a large Italian family. The defect is the result of an Alu element insertion.

Alu sequences are the most frequent, short, interspersed elements in the human genome, numbering 1.1 million copies and more than 10% of the mass of the genome. Alu elements are sequences of approximately 300 nucleotides flanked by perfect direct repeats of the host sequence, consistent with insertion into staggered nicks in the target DNA [8]. Alu and other repetitive elements have had great influence on the human genome and its evolution [9]. They influence mRNA splicing, RNA editing, and protein translation [10]. Not surprisingly, Alu insertions have been associated with several human genetic disorders. A total of 26 L1 trans-driven Alu insertions have so far been described as causes of diseases [11].

In our case the Alu element was inserted in the antisense direction with respect to *OPA1*. Alu element insertions in introns can have strong effects on gene expression, especially when inserted in the antisense direction. Five reports indicate that Alu insertions into intronic sequences in antisense orientation and close to the affected exon (19–50 nucleotides) cause the downstream exon to shift from constitutive splicing to full exon skipping or alternative splicing. Three have been found to cause skipping of downstream exons: one impairs correct recognition of the splice acceptor site (*F8* gene) [12], while the other two may affect the branch site that is usually very close to the end of the intron (*NF1* and *TNFRSF6* genes) [13,14]. The orientation and position of the Alu in the upstream intron was recently studied. Lev-Maor et al. shed light on the positive and negative effects of Alu elements inserted in antisense orientation immediately upstream of the regulated exon [15].

In our case Alu insertion probably interferes with the branch site leading to production of an alternatively spliced mRNA lacking exon 8. Loss of this exon may be similar to that reported for Alu element insertion in *NF1* and *TNFRSF6* genes.

A consensus target site is probably generated in the antisense strand where integration of the retroposon begins: the first 5′-gtAAAA-3′ hexanucleotides (3′CA↓TTTT 5′) of the 17-bp target-site duplication flanking the Alu element matches one of the more frequent consensus sequences perfectly [16]. The sense strand is nicked 17 bp from gtAAAA [17].

Phenotypic expression of this new mutation did not show particular peculiarities since ophthalmologic variability observed in our patients has also been described in other common *OPA1* mutations. Moreover, the presence of the mutation in two unaffected family members suggests incomplete penetrance, as previously reported for other *OPA1* gene mutations [18].

In conclusion, we describe an AluYb8 [8] retrotransposition event in an intron of the *OPA1* gene leading to exon skipping. The predicted consequence of this mutation is the loss of the GTPase activity of OPA1. Alu insertions have been reported in the literature as causing human genetic disease. However, this is the first report of a pathogenic *OPA1* gene mutation resulting from an Alu insertion.
